# Clinicopathological Study of Placentae of Twin Pregnancies with Emphasis on Exploration of Expression of Two Novel Markers, Annexin A5 and Apelin

**DOI:** 10.5146/tjpath.2026.14668

**Published:** 2026-09-15

**Authors:** Senjuti Dasgupta, Uma Banerjee, Partha Mukhopadhyay, Biplab Das, Saswata Saha

**Affiliations:** Department of Pathology, Medical College, WEST Bengal, İndia; Department of Pathology, Medical College, West Bengal, India

**Keywords:** Annexin A5, Apelin, Clinicopathological features, Placenta, Twins

## Abstract

Objective: Twin pregnancies are accompanied by various perinatal complications. The study of placentae is important for understanding the underlying pathogenesis and pathologies. Annexin A5 (ANXA5) is an anti-thrombotic agent, which is expressed by the placental trophoblasts. Apelin is a novel adipokine with a significant role in angiogenesis of the placenta.

The aim of the present study was to compare the gross, histopathological findings and immunohistochemical expression of ANXA5 and apelin of twin placentae with those of matched controls. These findings were also correlated with the clinical features of both mother and neonate.

Material and Methods: A prospective, observational study was undertaken for a span of one year. Twin placentae were collected along with their matched controls. The gross and microscopic features, as well as immunohistochemical expression of ANXA5 and apelin in these placentae were noted.

Results: There was a significantly lower expression of ANXA5 and apelin in the 24 twin placentae collected when compared with matched controls. The histopathological features of twin placentae included villous infarction (8, 33%), fibrin deposition (24, 100%), chorangiosis (18, 75%), and intraplacental hematoma (18, 75%).

Conclusion: Both ANXA5 and apelin are vital for placental homeostasis. The reduction in expression of these markers in twin pregnancies indicates their contribution to adverse outcomes of these cases. Studies with larger sample size are required in the future for further exploration of the role of these novel markers.

## Introduction

In recent times, there has been an increase in the incidence of twin pregnancies worldwide. This rise may be attributed to various reasons including advanced maternal age, increased use of fertility treatments, advances in reproductive technologies, and various genetic and environmental factors (1).

Twin pregnancies may be monochorionic or dichorionic. The risk of complications in case of twin pregnancies is more than the singleton ones, and that of monochorionic twins is greater than the dichorionic twin pregnancies. These include hypertensive disorders of pregnancy, preterm labour, postpartum hemorrhage, prematurity and low birth weight. There is increased perinatal morbidity and mortality among the twins (2).

Placental examination in case of twin pregnancies holds promise to yield valuable information regarding the underlying pathogenesis and pathologies. The approach to examination of placentae has been standardized in recent times, which, when followed, ensures their meticulous study (3).

Since the twin pregnancies are so frequently accompanied by varied complications, it has been a matter of active research to find out the various factors that are responsible for ensuring placental health in these cases. Annexin A5 (ANXA5) and Apelin are molecules that play very important roles in maintaining placental homeostasis. ANXA5 belongs to the family of 12 structurally related, highly conserved annexin proteins. Studies reveal that ANXA5 prevents inappropriate coagulation in the placenta, thereby preventing fetal loss. This molecule is extensively expressed on the surface of syncytiotrophoblasts in normal human placentae (4,5). Apelin is a peptide that acts as an adipocytokine and also executes cardiovascular functions. It has been known to play a vital role in placental angiogenesis. Apelin is mostly produced in the lungs but it is also known to be synthesized in the placenta, where it has a paracrine role (6–8).

Despite the established roles of ANXA5 and apelin in placental physiology, their combined evaluation in the context of twin pregnancies has not been adequately explored. To date, there are limited comparative studies evaluating both ANXA5 and Apelin expression in twin placentae. Previous studies have largely focused on their expression in singleton pregnancies or in specific maternal complications such as pre-eclampsia and gestational diabetes. As a result, there is a clear knowledge gap regarding how these two key regulatory molecules behave together in the unique physiological environment of twin gestations, which are inherently associated with increased placental demand and stress.

The scientific rationale for the present study therefore arises from the need to understand whether the altered placental environment in twin pregnancies affects the expression of ANXA5 and apelin, individually and collectively, and whether these changes may contribute to adverse neonatal outcomes. To address this gap, the study was designed with the hypothesis that twin placentae show altered expression of ANXA5 and apelin compared with singleton placentae, and that these alterations may correlate with clinical and neonatal parameters. This represents a novel approach, as no prior studies have simultaneously examined both markers in twin placentae.

The objectives of the present study were to study the gross and histopathological features of the placentae of twin pregnancies and to compare them with those of control placentae. The study also aimed to study the immunohistochemical expression of ANXA5 and apelin in twin placentae and to find out if there is any correlation between the level of expression of these markers and neonatal health.

## Materials and methods

A prospective observational study was undertaken in the Department of Pathology in close collaboration with the Gynaecology Department in a tertiary care centre of Eastern India, following approval of the Institutional Ethical Committee. The duration of the study was 11 months, from September 2023 to July 2024, rather than a full calendar year, for better precision. All the mothers who delivered twin babies in the institute during this time period were the target population of the study. A patient consent form was provided to each of the mothers in the vernacular and all aspects of the study were explained to them in details.

The inclusion criteria were mothers with twin pregnancies who underwent their deliveries in the institute during the study period. Only those who voluntarily agreed to participate in the study and signed the consent form were included in the study. The exclusion criteria were mothers with singleton pregnancies and those with twin pregnancies who refused to provide consent to take part in the study. There were 24 twin pregnancy deliveries in the institute during the time period of the study and all these cases were included.

The sample size was determined using the standard formula z2pq/d2. In this formula, z is the value of standard normal distribution corresponding to a significance level of 0.05 (z = 1.96), p is prevalence of twin pregnancies in India (p = 3.2%), q is (1-p), and d is allowable error (10%) (9). The minimum sample size was calculated to be 11, according to this formula.

The placentae were collected in 10% neutral buffered formalin following delivery of the mothers of twin pregnancies. The placentae of matched controls (singleton pregnancies) were also simultaneously collected. Control placentae were matched to twin cases based on gestational age (±1 week), maternal age (±2 years), parity, and absence of more than one major obstetric complication. All relevant clinical details of the cases were noted including gestational age and birth weight of the babies. Gross examination of the collected placentae was done to note their shape, diameter, thickness, weight, nature of umbilical cord attachment, and number of cotyledons. Special attention was given to the details of the dividing membrane in each case.

The sections that were submitted for histopathological examination included those taken from the umbilical cord, area of cord insertion, and membranes and at least 3 full thickness sections were obtained from the placental parenchyma, both from apparently normal and abnormal looking areas. Two pathologists examined the haematoxylin and eosin (H&E) stained slides. The findings of the two pathologists were later compared, and in case of disagreement the slides were re-examined by both of them to arrive at a consensus.

Immunohistochemical analysis (IHC) was performed on full thickness placental tissue sections with ANXA5 and Apelin antibodies. The ANXA5 clone orb36728 (Biorbyt, UK; Lot number – E5889) was used at a dilution of 1:100. The positive control in this case was hepatocellular carcinoma sections. The apelin clone orb247041 (Biorbyt, UK; Lot number – BS9103) was used at a dilution of 1:200. Human glial tissue was the positive control used for the apelin antibodies. Omission of the primary antibodies served to provide the negative controls in both cases. Sections were deparaffinized, rehydrated, and subjected to heat-induced antigen retrieval in citrate buffer (pH 6.0) at 95–98°C for 20 minutes. Endogenous peroxidase activity was blocked with 3% hydrogen peroxide for 10 minutes. Primary antibodies were incubated at room temperature (22–25°C) for 60 minutes, followed by incubation with secondary antibody for 30 minutes at room temperature. DAB chromogen was applied for 5 minutes and slides were counterstained with haematoxylin for 1 minute. The IHC analysis was once again done by two pathologists independently.

The expression of the two markers, ANXA5 and apelin, was scored with respect to their extent and intensity. “Hot spots” were identified under low power and then 300 to 500 cells were examined in 10 high power fields using x400 magnification. The percentage of positive cells was assigned the following scores: score 0 for no immunoreactive cells, score 1 for ≤ 10% positive cells, score 2 for 10 to 50% positive cells, score 3 for 51 to 80% positive cells, and score 4 for >80% positive cells. The intensity of staining was scored as 0.5 for very weak staining, 1 for weak staining, 2 for mild staining, and 3 for strong staining (10). When the two pathologists who examined the cases had discrepant scores, they re-examined the slides together in order to reach a consensus. Both pathologists achieved complete agreement after consensus review.

Tabulation of all the findings was done meticulously. Normally distributed continuous variables were expressed as mean and range. These variables were compared between the twin pregnancies and control groups using unpaired t test-two tailed. Before applying parametric tests, normality of continuous variables was assessed using the Shapiro–Wilk test. Categorical variables were expressed as percentages, and compared between the groups using either Fischer’s exact test or Chi square test. All statistical analyses were done using GraphPad Prism 6™. A p value < 0.05 was considered to be statistically significant.

## Results

A total of 24 placentae were collected following deliveries of twins between September, 2023 and July, 2024. Pregnancy-induced hypertension was noted in 8 (33.3%) mothers, placenta previa in 4 (16.7%), and gestational diabetes mellitus in 2 (8%). Out of these 24 cases, 14 (58.3%) were dichorionic and 10 (41.7%) were monochorionic twin pregnancies. Preterm births were significantly more common among the twin pregnancies, when compared with the control group (p = 0.000266). The number of babies born with low birth weight and those requiring treatment in the sick neonatal care unit (SNCU) was also significantly higher among the twin group (p<0.00001 in both cases). A significant correlation was also noted between chorionicity and requirement of SNCU treatment at birth(Table 1).

**Table 1:** Comparison of clinical features between twin and control group of patients and between monochorionic and dichorionic groups of twin pregnancies

**Clinical features**	**Comparison between Twin and Control groups**		**p value**	**Comparison between Monochorionic and Dichorionic**		**p value**
	**Twin,** **n=24 (100%)**	**Control, n=24 (100%)**		**Dichorionic, n=14 (100%)**	**Monochorionic, n=10 (100%)**	
**Term vs. Preterm**						
Term	12 (50)	24 (100)	**<0.001**	6 (43)	6 (60)	0.407626
Preterm	12 (50)	0		8 (57)	4 (40)	
**Birth weight**						
Normal Birth weight	2(8)	24 (100)	**<0.001**	2(14)	0	0.691545
Low Birth weight	22(92)	0		12(86)	10(100)	
**Resuscitation**						
Spontaneous cry at birth	21 (87)	24 (100)	0.277339	13 (93)	8 (80)	0.347755
Resuscitation required at birth	3 (13)	0		1 (7)	2 (20)	
**Treatment at SNCU**						
Treatment not required	8 (33)	24 (100)	**<0.001**	12 (85.7%)	4 (40)	**0.019172**
Treatment required	16 (67)	0		2 (14.3%)	6 (60)	

** SNCU: **Sick neonatal care unit.

The mean placental weight was 562±113 grams (the range being 300 to 700 g) and the mean combined birth weight of the twin babies was 3.4±0.67 kg (range being 2.4 to 3.9 kg). The mean birth weight to placental weight ratio was 6.36±1.78. The values of mean placental weight when compared to those of control placentae (475±48.6 grams) were found to be statistically significant. (p=0.0012) The ratio of birth weight to placental weight was also found to be significantly different from that of the control group (5.6±0.3; p=0.04). The insertion of one of the umbilical cords in the twin placentae was found to be marginal in 6 cases (25%) and velamentous in 2 (8.3%).

**Figure 1:**
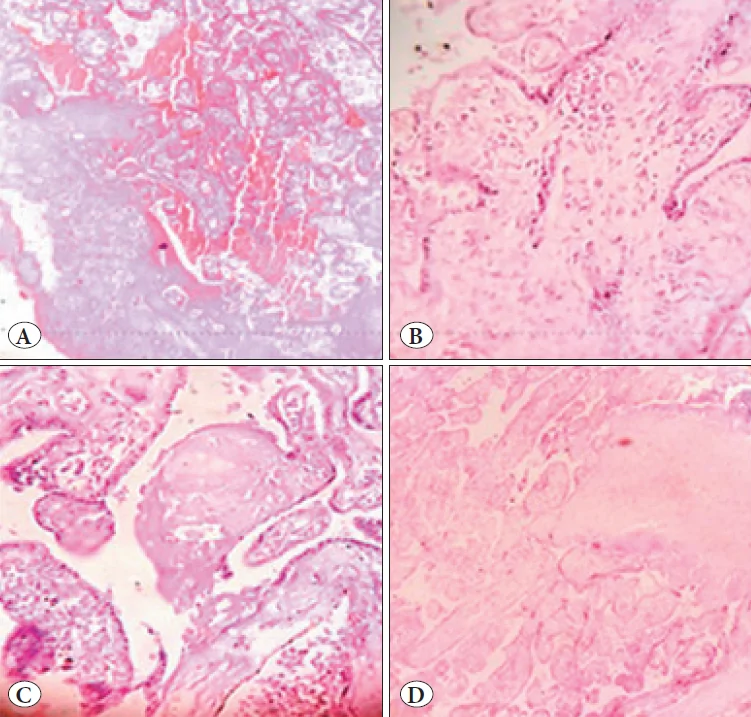
Sections from twin placenta showing the following features – A) Intraplacental hematoma (H&E, x100), B) chorangiosis (H&E, x100), C) fibrin deposition (H&E, x100), D) villous infarction (H&E, x100)

Microscopic examination of the twin placentae revealed the following lesions: villous infarction in 8 cases (33%), fibrin deposition in all 24 cases (100%), and chorangiosis and intraplacental hematoma in 18 cases each (75%). Infarction was diagnosed by the recognition of one or more of the following microscopic features: intravillous haemorrhage, congestion of villous capillaries, collapse of intervillous spaces, villous agglutination, smudging of nuclei, loss of nuclear basophilia of syncytium, ghost villi, pyknosis, and karyorrhexis (11). Chorangiosis was diagnosed when there were more than 10 capillaries in more than 10 terminal chorionic villi in numerous areas of the placenta (12). All the microscopic features showed statistically significant association with placentae of twin pregnancies when compared with the control placentae (Table 2, Figure 1).

**Table 2:** Comparison of histopathological features of placenta between twin and control groups

**Histopathological features**	**Comparison between Twin & Control groups**		**p value**
	**Twin,** **n = 24 (100%)**	**Control,** **n =24 (100%)**	
**Chorangiosis**			
Present	18(75)	0	**<0.001**
Absent	6(25)	24 (100)	
**Fibrin deposition**			
Present	24(100)	0	**<0.001**
Absent	0	24 (100)	
**Intraplacental hematoma**			
Present	18 (75)	0	**<0.001**
Absent	6 (25)	24 (100)	
**Villous infarction**			
Present	8(33)	0	0.008029
Absent	16(67)	24 (100)	

**Figure 2:**
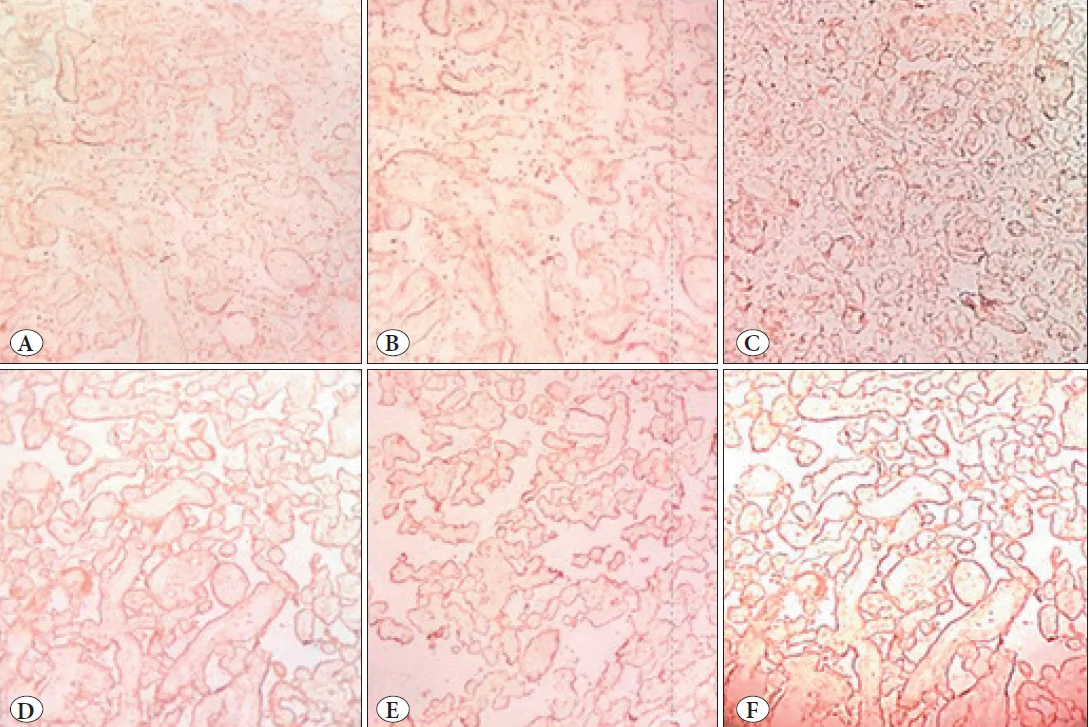
Annexin A5 expression in twin placentae: Intensity of expression – A) 1, B) 2, C) 3, (Immunohistochemical examination, x100); Apelin expression in twin placentae: Intensity of expression – D) 1, E) 2, F) 3, (Immunohistochemical examination, x100).

IHC analysis revealed that there was a statistically significant difference in the expression of ANXA5 and apelin, both in terms of grade and intensity, between the twin and control placentae (Table 3). The expression of ANXA5 was maximum in the syncytiotrophoblasts and that of apelin in the villous stroma of the twin placentae (Figure 2). The expression of these markers was also correlated with chorionicity and clinical features. A statistically significant association was found among the twins who required SNCU treatment, and showed greater intensity of expression of apelin in dichorionic twins than the monochorionic ones.

**Table 3:** Placental expression of ANXA5 and Apelin in terms of both grade and intensity – comparison between twin placentae and matched controls

	**No. (%)**	**Grade**				**p value**	**Intensity**			**p value**
		**G-1**	**G-2**	**G-3**	**G-4**		**I-1**	**I-2**	**I-3**	
**ANXA5**						**0.001**				**0.0011**
Twin, n (%)	24 (100)	8 (33.3)	5 (20.8)	8 (33.3)	3 (12.5)		6 (25)	8 (33.3)	10 (41.7)	
Control, n (%)	24 (100)	1 (4.2)	1 (4.2)	6 (25)	16 (66.7)		1 (4.2)	1 (4.2)	22 (91.7)	
**Apelin**						**<0.001**				**<0.001**
Twin, n (%)	24 (100)	16 (66.7)	3 (12.5)	4 (16.7)	1 (4.2)		17 (70.8)	5 (20.8)	2 (8.3)	
Control, n (%)	24 (100)	1 (4.2)	1 (4.2)	8 (33.3)	14 (58.3)		1 (4.2)	1 (4.2)	22 (91.7)	

## Discussion

Twin pregnancies are at a higher risk of perinatal complications compared to singleton pregnancies. The major problems that may arise are pre-eclampsia, antepartum and postpartum haemorrhage, miscarriages, stillbirths, preterm deliveries and intra-uterine growth restriction. Babies born from twin pregnancies are susceptible to respiratory disorders, developmental anomalies and seizures (13). In the present study, pre-eclampsia was noted in 8 (33.3%) mothers, placenta previa in 4 (16.7%), and gestational diabetes mellitus (GDM) in 2 (8%). Similar maternal complications of twin pregnancies have been reported in other studies. (14,15) The current study recorded a significantly higher number of preterm births, low birth weight babies, and those requiring SNCU treatment among the twin pregnancies compared to the singleton ones. These findings were corroborated with other studies (2,16). In the present study, there was a significantly higher number of monochorionic twin babies who required SNCU treatment when compared with the dichorionic ones. Chorionicity is related with both perinatal and neonatal outcome and the dichorionic pregnancies have a favourable prognosis over the monochorionic ones. This is mostly attributed to the sharing of a single placenta and earlier gestational age at birth in case of monochorionic twins (17).

A velamentous insertion of one of the umbilical cords is 8 times more common in twin placentae compared to the singleton ones (13). The present study recorded velamentous insertion of one of the umbilical cords in 2 (8.3%) cases and marginal insertion in 6 (25%). There was statistically significant difference in mean birth weight to placental weight ratios in the twin pregnancy group when compared to the control group. De Paepe et al. have considered birth weight to placental weight ratio to be an index of functional efficiency of the placentae (18).

Histopathological examination of the placenta is essential to understand the different lesions underlying the various complications that accompany twin pregnancies. The various lesions found in twin pregnancies include villous infarction, villous immaturity, chronic villitis, fibrin deposition, chorangiosis, and intraplacental hematoma (19). In the present study, the various microscopic lesions were found to be significantly related with twin placentae compared to singleton ones. These features were also more commonly found in monochorionic twins than the dichorionic ones, even though the difference was not statistically significant. These findings were comparable to those reported by Hubinont et al. in their study (13).

ANXA5 and apelin are novel markers and a thorough review of literature has revealed that the placental expression of these markers in twin placentae has not been explored extensively, till date. These markers are relevant since they have an indisputable role in ensuring placental homeostasis. It has been repeatedly stressed that the substances that are important regulators of placental health need to be studied in detail to explore the different paths for improving the course of complicated pregnancies (20). These two molecules, ANXA5 and apelin, have emerged over the years as very important candidates for such exploration.

In the present study, a statistically significant decrease in the expression of ANXA5 and apelin in the placentae of twin pregnancies, was noted both in terms of grade and intensity, when compared with the singleton pregnancies. Since there were 14 cases (58.3%) in the study, which were complicated by other conditions (pre-eclampsia, GDM, placenta previa) as well, it was a matter of concern to delineate if the difference could be attributed to twinning alone. The authors are of the opinion that the other conditions had a synergistic effect in reducing the expression of the two markers in the current study. This is because the cases, which were not further complicated by other conditions (10, 41.7%), also showed similar results. However, the observed decrease in ANXA5 and apelin expressions is likely multifactorial, and the relative contribution of twinning versus maternal complications requires further investigation with larger cohorts.

ANXA5 is vital in several cellular processes including mineralization of cartilage, intracellular signalling and inhibition of phospholipase A2 and protein kinase C. ANXA5 binds to phosphatidylserine on vascular endothelial cells in the placenta and assists in the suppression of inappropriate blood coagulation during pregnancy (4). Apelin is the product of the APLN gene. It is an endogenous ligand of a G protein-coupled receptor called APJ. APJ is intimately associated with angiotensin receptor (AT1). The apelin/APJ complex plays an important role in cardiovascular pathophysiology. It functions as a potent inotropic agent and causes endothelium-mediated vasodilation. Apelin is believed to have a paracrine role in human chorionic villi as it has been identified in the syncytiotrophoblasts, cytotrophoblasts, and foetal endothelial cells (7). These pathophysiologic roles of ANXA5 and apelin provide plausible explanations for the association of reduced levels of these molecules with placental dysfunction including hypoxia, coagulation imbalance, and vascular insufficiency.

Hypoxia in twin placentae can downregulate both ANXA5 and apelin, contributing to villous maldevelopment and impaired nutrient exchange. Decreased ANXA5 may also predispose to pro-coagulant states within the intervillous space, resulting in fibrin deposition and microthrombi, ultimately worsening placental dysfunction. Similarly, reduction in apelin may reflect compromised vascular signaling, impaired angiogenesis, and dysregulated vasodilation, all of which are crucial for maintaining optimal placental perfusion.

Given their consistent reduction in complicated twin pregnancies, ANXA5 and apelin may have potential as early predictive biomarkers of placental insufficiency. Their biological roles in membrane stabilization, vascular regulation, and inflammatory control also highlight their promise as possible therapeutic targets (21).

Colcimen et al. noted a significant reduction of ANXA5 in pre-eclamptic placentae (10). Oliva et al. reported reduction of ANXA5 in cases of GDM (22). On the contrary, an increase in the expression of ANXA5 has been reported in GDM placentae by Liu et al. (23). ANXA5 is claimed to be of utmost importance in maintaining overall integrity of placenta and fluidity in the intervillous compartments (10).

The expression of apelin has been reported to be reduced in pre-eclamptic placentae by Van Mieghem et al. and Yamaleyeva et al. (7,24). Inuzuka et al. also noted fall in apelin expression in the placentae of patients with severe pre-eclampsia (6). However, a rise in apelin levels was found in pre-eclampsia by Colcimen et al. and Cobellis et al. (10,25). Oncul et al. reported a fall in apelin levels in GDM patients (26). Van Mieghem et al. also reported a strong reduction of apelin staining in syncytiotrophoblasts and villous stroma of placentae in cases of intrauterine growth retardation (7).

An extensive search of the literature makes it apparent that research work with respect to the two molecules, ANXA5 and apelin, is still at the incipient stage. The results of various studies need to be reviewed in the light of confounding factors in order to draw pertinent conclusions regarding the contradictory results obtained in some cases of complicated pregnancies. The present study is limited by its small sample size. The comparison between monochorionic and dichorionic twin placentae did not reveal much difference except for significantly higher intensity of apelin expression among dichorionic placentae of babies who required SNCU treatment compared to those with monochorionic placentae. The authors are of the opinion that this could have been an incidental finding. Since the sample size was small, true comparison between monochorionic and dichorionic twin placentae was not feasible in the present study. Further studies with larger samples are required in future to continue exploration of these two molecules, ANXA5 and apelin, as they hold a lot of promise in unravelling the placental mysteries of various complications that commonly accompany pregnancies. The limitations of the study may be addressed by future multicentric and longitudinal studies to validate the biomarker roles of ANXA5 and apelin and to overcome sample-size constraints.

## Conclusion

Placental examination is of utmost importance for studying the intricacies involved in complicated pregnancies. The twin pregnancies have their own share of complications and these are further enhanced by the fact that they are frequently accompanied by other conditions like pre-eclampsia. The present study unravelled the various gross and microscopic characteristics of the twin placentae. The correlation between twin pregnancies and various clinicopathological features was also explored in the study. The two research molecules, ANXA5 and apelin, are crucial for placental health. The expression of these markers is being explored in various types of complicated pregnancies in different studies, worldwide. The reduced expression of ANXA5 and Apelin in twin placentae indicates their potential involvement in the adverse outcomes of twin pregnancies. Larger, multicentric studies are warranted to validate these findings.

## Conflict of Interest

The authors declare that there is no conflict of interest.
